# Perinatal characteristics among early (10–14 years old) and late (15–19 years old) pregnant adolescents

**DOI:** 10.1186/1756-0500-5-531

**Published:** 2012-09-25

**Authors:** João Guilherme Bezerra Alves, Rosangela Meira Rodrigues Cisneiros, Luciana Paula Fernandes Dutra, Renato Américo Pinto

**Affiliations:** 1 Instituto de Medicina Integral Prof. Fernando Figueira (IMIP), Rua dos Coelhos 300, Boa Vista, Recife PE, ZIP: 50070-550, Brazil; 2 Universidade do Vale do São Francisco (UNIVASF), Av. José de Sá Manisoba, s/n Campos Universitário, Petrolina, PE, 56304-205, Brazil; 3 Faculdade Pernambucana de Saúde (FPS), Av. Jean Emile Favre, 422, Imbiribeira, Recife, PE, 51200-060, Brazil

**Keywords:** Pregnancy, Adolescent, Low birth weight, Prematurity, Socioeconomic condition

## Abstract

**Background:**

Pregnancy in adolescents is a worldwide health problem and has been mostly common in poor populations. It is not clear if socioeconomic or biological factors are the main determinants of perinatal adverse outcomes in pregnant adolescents. Adolescents under 15 years old may present a high growth rate which may contribute to impair fetal growth. Our aim is to compare perinatal characteristics among early (aged 10 to 14 years) and late (aged 15 to 19 years) pregnant adolescents.

**Methods:**

A cross-sectional study was performed using data from Pernambuco State 2009, obtained from DATASUS/SISNAC, a Brazilian Government, open-access public health database. Maternal and neonatal outcomes were compared between early (aged 10–14 years) and late (aged 15–19 years) pregnant adolescents. Family income was compared between early and late pregnant adolescents using a sample of 412 subjects evaluated at Instituto de Medicina Integral Prof. Fernando Figueira (IMIP) during 2011. Statistical comparisons were made using the chi-square test was used with a significant level of 0.05; bivariate and multivariate analysis were performed. This project was approved by the Institutional Ethics Review Board.

**Results:**

Data from 31,209 pregnant adolescents were analyzed. 29,733 (95.2%) were aged 15 to 19 years and 1,476 (4.7%) were aged 10 to 14 years. There were significant differences with respect to marital status, education level and number of prenatal visits of mothers aged 10 to 14 years compared to 15 to 19 years. Of importance, early adolescents had a greater rate of neonates born premature and with low birth weight. Prematurity and low birth weight remained statistically significant after multivariate analysis.

**Conclusions:**

Early aged adolescents may have an increased risk of prematurity and low birth weight. These findings highlight the potential role of biological factors in newborn outcomes in pregnant adolescents.

## Background

Adolescent pregnancy is a worldwide health problem especially relevant in developing countries [1,2]. It is associated with an increased risk of adverse maternal and fetal outcomes such as maternal and neonatal mortality, cesarean section, preterm birth and low birth weight [2,3]. These poor outcomes may be explained by a possible physical and psychological immaturity for reproduction in adolescents [1]. In addition, adolescents usually have adverse social-economic factors that may affect the outcome of pregnancy [3,4].

However, it is unclear whether biological or socio-economic factors are more important for the adverse outcomes in the pregnant adolescent. For some researchers, biological factors such as age or maternal growth are not a risk factor, and unfavorable outcomes are more likely associated with socio-economic and lifestyle factors [5-7]. Indeed pregnant adolescents usually have socio-economic disadvantages, less schooling and little social support [8]. On the other side, some studies have found that adolescents have more adverse pregnancy outcomes as compared to adult women, even after controlling for socio-economic factors [9-11].

Most studies in the pregnant adolescent focus on adolescents aged 15 to 19 years whereas information about pregnancy at younger ages usually appears only in aggregate statistics [12-14]. Adolescents under 15 years may have peculiar biological characteristics such as higher growth rate, more intense hormonal changes, less developed muscles, joints and bones, especially the pelvis, which may contribute to impair fetal growth and lead to low birth weight. Despite this, only a few studies have evaluated whether pregnancy outcomes is different among early (aged 10–14 years) and late (aged 15–19 years) adolescents. Our aim is to compare birth outcomes among early and late pregnant adolescents in a developing country such as Brazil.

## Results

In Pernambuco State, 31,209 live births from teenage mothers were registered during 2009 were registered; 29,733 (95.2%) were aged from 15 and 19 years and 1,476 (4.7%) aged from 10 to 14 years.

The maternal characteristics were different among early and late pregnant adolescents regarding marital status, education level and number of prenatal consultations (Table 1). There were no differences in family income between early and late pregnant adolescents, with US $321,00 vs US $319,00 per month, respectively (p = 0.85). Newborns showed differences in birth weight (lower in early adolescents), preterm birth (higher in early adolescents) and Apgar score (lower in early adolescents). (Table 2)

**Table 1:** Characteristics of early and late pregnant adolescents

	**10 – 14 years 1476 (%)**	**15 – 19 years 29733 (%)**	p *
Single mothers	1453 (98.4%)	26256 (88.3%)	<0.0001
Cesarean delivery	504 (34.2%)	10473 (35.2%)	0.39
>7 prenatal attendance	541 (47.6%)	12180 (40.9%)	0.001
>8 years schooling	204 (13.8%)	12411 (41.7%)	0.0001
White color	330 (22.4%)	7616 (25.7%)	0.005
Hospital delivery	1458 (98.7%)	29383 (98.8%)	0.88

* Chi-square test.

**Table 2:** Neonatal characteristics among early and late pregnant adolescents

	**Aged 10 – 14 years 1476 (%)**	**Aged 15 – 19 years 24733 (%)**	p *
Low birth weight	209 (14.1)	2628 (8.8)	0.0001
Preterm birth	154 (10.4)	1835 (6.1)	0.0001
Male	791 (53.5)	15194 (51.1)	0.06
Apgar > 7 (5’)	1150 (77.9)	24105 (81.0)	0.002
Malformation	11 (0.7)	225 (0.7)	0.96

* Chi-square test.

Lower birth weight (crude odds ratio: 3.0; 95%CI 2.8 – 3.2; adjusted odds ratio: 3.5; 95%CI 3.2 – 3.8) and preterm birth (crude odds ratio: 1.6; 95%CI 1.4 – 1.9; adjusted odds ratio: 1.6; 95%CI 1.4 – 1.9) remained statistically significant after multivariate analysis, but not Apgar score (crude odds ratio: 1.2; 95%CI 1.0 – 1.3; adjusted odds ratio: 0.9; 95%CI 0.8 – 1.1).

## Discussion

Early pregnant adolescents in Pernambuco, Brazil, in 2009, gave birth to neonates with lower birth weight and also had a higher rate of preterm births, as compared with late pregnant adolescents. In Brazil, teenage pregnancy is more common among lower income adolescents and in Pernambuco state over 90% of births are registered at public hospitals [15]. Importantly, the sample of early and late pregnant adolescents attended at IMIP showed no differences in family income suggesting that biological, rather than socio-economic factors, play a larger role in the adverse neonatal outcomes seen in these early pregnant adolescents. However our data are observational and causation should not be inferred. Beyond we had used a national database and socio-economic variables cannot be well controlled. Some studies suggests that a risk factor for adverse birth outcomes are associated with disadvantaged environments and poor behavioral choices that are more commonly seen in younger teenage mothers [3,4].

The influence of biological factors on pregnant adolescent outcomes has not been adequately studied in humans. Birth weight, for example, depends more on environmental than genetic factors [16]. A limited uterine space, which may be more associated with early than late adolescents, can impair fetal growth [17]. Also, the early adolescent body growth rate may further contribute to diminish nutrient availability to the fetus further limiting fetal growth.

Our findings are consistent with others studies. Conde-Agudelo et al. [18] found that in Latin America rates of low birth weight and preterm birth consistently increased with decreasing maternal age and were highest among infants born of mothers aged 15 years or younger. Olausson et al. [19] reported an increase in preterm birth among younger teenagers and suggested that young maternal age may be a biologic risk factor for preterm birth. Kurth et al. in Africa found that the probability to deliver an infant with low birth weight was more than doubled for adolescent below 16 years [20].

However there are some discordant findings. Magalhães et al. [21] found differences in neonatal vitality among early and later pregnant adolescents but not in birth weight and preterm birth. Hoque & Hoque [22] did not find any additional risks of pregnancy outcomes among early adolescent pregnancies. Lubarsky et al. [23] found the same frequency of birth weight and preterm birth among teenagers aged below 15 and mothers aged 20–29. A possible explanation for the discrepancy between our findings and those cited above may relate to the fact that these studies considered early adolescents aged below 16 and not aged 10 to 14.

Our study has some limitations. First of all we studied a secondary data provided by the Brazilian Government through DATASUS/SINASC. However, these data have been widely used and the health information system in Brazil has been more rigorous and precise in their collecting and dissemination. Secondly, we could not control for other variables that contributes to low birth weight, preterm birth and neonatal vitality. Nevertheless, smoking and alcohol consumption in adolescents increase with age at adolescence, and primiparity is associated with lower birth weight but not with preterm birth. It should be added that we have studied a large number of adolescents and we found a strong difference among neonatal outcomes.

## Conclusions

Our findings suggest that early adolescent pregnancy may increase the risk of low birth weight and preterm birth. Biological maternal characteristics of an early pregnant adolescent may be more important than socio-economic factors for adverse outcomes in adolescent pregnancy.

## Methods

This cross-sectional study analyzed data from DATASUS/SINASC database ( http://www.datasus.gov.br), a national open-access public health system database, organized and maintained by the Brazilian government. 141,815 records of live births were observed and 31,209 (22.0%) teen mothers (aged 10 to 19 years) were studied in Pernambuco State during 2009.

Early adolescents were defined those aged from 10 to 14 years, and late adolescents those aged 15 to 19 years. The following variables were studied: age, race, marital status, schooling, type of delivery and birth place, gender of newborn, birth weight, preterm birth, neonatal hypoxia and malformations. Low birth weight was defined as a live infant weighting < 2500 g at birth, preterm birth as a live infant delivered at <37 weeks’ gestation and neonatal anoxia as an Apgar score < 7 at 5'.

To asses family income, a questionnaire was administered to a sample of 412 pregnant adolescents (22 early and 390 late) seen from March to December, 2011, at Instituto de Medicina Integral Prof Fernando Figueira (IMIP), Recife, Pernambuco.

The number of inhabitants from Pernambuco State was obtained from the IBGE - The Brazilian Institute of Geography and Statistics (15).

Ethics approval was obtained from the Ethics Review Boards at IMIP. A descriptive analysis of the data was performed through frequency tables with numbers and percentages. Socio-economic and biological characteristics among early and late pregnant adolescents were compared. Chi-square test was used with a significance level of 0.05. Bivariate and multivariate analysis were performed using the Statistical Package Social Science (SPSS) Version 15 was used.

## Competing interests

The authors declare they have no competing interests.

## Authors’ contributions

Each author has participated sufficiently and actively in this study. JGA conceived and coordinated the study, drafted the manuscript; RMC contributed to the data collection and made a substantial contribution to analysis and interpretation; LPD also contributed to the data collection and the editing of the manuscript; RAP has participated in the design of the study and performed the statistical analysis. Each author revised the manuscript and provided the final approval of the version to be published.
